# Microarray Approach Combined with ddPCR: An Useful Pipeline for the Detection and Quantification of Circulating Tumour DNA Mutations

**DOI:** 10.3390/cells8080769

**Published:** 2019-07-24

**Authors:** Silvia Galbiati, Francesco Damin, Lucia Ferraro, Nadia Soriani, Valentina Burgio, Monica Ronzoni, Luca Gianni, Maurizio Ferrari, Marcella Chiari

**Affiliations:** 1 Genomic Unit for the Diagnosis of Human Pathologies, Division of Genetics and Cell Biology, IRCCS San Raffaele Scientific Institute, 20132 Milan, Italy; 2 Istituto di Chimica del Riconoscimento Molecolare, Consiglio Nazionale delle Ricerche, 20131 Milan, Italy; 3 Dipartimento di Oncologia Medica, IRCCS Ospedale San Raffaele, 20132 Milan, Italy; 4 Laboratory of Clinical Molecular Biology, IRCCS Ospedale San Raffaele, 20132 Milan, Italy; 5 Università Vita-Salute San Raffaele, 20132 Milano, Italy

**Keywords:** ctDNA, liquid biopsy, metastatic colorectal cancer, microarray, ddPCR

## Abstract

It has now been established that in biological fluids such as blood, it is possible to detect cancer causing genomic alterations by analysing circulating tumour DNA (ctDNA). Information derived from ctDNA offers a unique opportunity to enrich our understanding of cancer biology, tumour evolution and therapeutic efficacy and resistance. Here, we propose a workflow to identify targeted mutations by a customized microarray-based assay for the simultaneous detection of single point mutations in different oncogenes (*KRAS, NRAS* and *BRAF*) followed by droplet digital PCR (ddPCR) to determine the fractional abundance of the mutated allele. Genetic variants were determined in the plasma of 20 metastatic colorectal cancer (mCRC) patients previously genotyped on tissue biopsy at the diagnosis for medication planning (T0) and following the tumour genetic evolution during treatment phase (T1 and T2) with the objective of allowing therapy response prediction and monitoring. Our preliminary results show that this combined approach is suitable for routine clinical practice. The microarray platform enables for a rapid, specific and sensitive detection of the most common mutations suitable for high-throughput analysis without costly instrumentation while, the ddPCR, consents an absolute quantification of the mutated allele in a longitudinal observational study on patients undergoing targeted therapy.

## 1. Introduction

Colorectal cancer (CRC) is the second most commonly diagnosed cancer in Europe and a leading cause of death both in Europe and worldwide [1,2]. In recent years there has been an increase in the diagnosis of CRC and a contextual decrease in mortality, due to screening programs, early diagnosis and improved therapies, which are increasingly targeted and personalized [3]. 

About 20–25% of patients with CRC have advanced disease at diagnosis and about 35% of patients treated with curative intent will develop an advanced disease.

The outcome for patients with metastatic CRC (mCRC) has been improved by targeting pathways involved in CRC development, such as epidermal growth factor receptor (EGFR) and vascular epidermal growth factor (VEGF) pathways [1,3,4].

Antiangiogenic drugs and EGFR inhibitors (the so-called target therapies) are placed next to the supporting skeleton represented by chemotherapy and their use in the management of patients affected by mCRC. Selecting the most appropriate first-line treatment is the first important issue in the management of mCRC, because this choice will guide subsequent treatments and also will define the treatment which will be able to achieve disease control, to obtain clinical benefit and to allow further loco-regional or systemic interventions [4].

For the use of molecular target therapies, it is mandatory that patients are selected based on the molecular profile of the tumour and in particular on the presence of mutations that indicate the possible response to treatment (predictive factor). 

*RAS* mutations are found in about 20–25% of solid tumours but in the setting of CRC this percentage rises to 40% [3].

The current recommendation is having *RAS* mutational status before starting first-line treatment for advanced disease, because it has been demonstrated that the benefit of anti-EGFR therapy is limited to patients in whom *RAS* mutation are excluded. 

In addition, it is essential to evaluate the *BRAF* mutational status: *BRAF* mutations (nearly always V600E) are found in 8–12% of patients with mCRC and this test has prognostic value because the *BRAF* mutation is a strong and widely accepted indicator of a poor prognosis related to the high biological aggressiveness of the disease, reduced response to standard therapies and early peritoneal spread [5,6,7].

Nowadays, for mCRC patients, the current standard of care for molecular testing is performed on tissue biopsy, however, it is now established that in biological fluids such as blood and in particular, in the plasma component, it is possible to detect cancer causing genomic alterations by analysing circulating tumour DNA (ctDNA). 

The most recently developed, highly sensitive, approaches suitable for mutation analysis in liquid biopsy are targeted next generation sequencing (NGS) and droplet digital PCR (ddPCR). However, several drawbacks limit their use in clinical practice. In particular, the high costs of the methodology restricts the usefulness of the deep sequencing. Costs matter if one considers that the main advantage of liquid biopsy is the non-invasiveness and thus the possibility to monitor the patients in several follow-up time points.

On the contrary, ddPCR is not an expensive technique, however, its major weakness is that a priori knowledge of the mutations to quantify is required. 

In this prospective pilot study, we proposed a combined pipeline in which a customized microarray-based assay approach enables the identification of the most frequent mutations involved in mCRC followed by a targeted ddPCR analysis to quantify the fractional abundance of the mutated allele as an useful and easily implementable workflow in tumour patient management. 

Indeed, we first assessed the possibility to use the microarray to discover correctly the mutations involved comparing the results obtained with those provided by tissue biopsy and then, when possible, we monitored patients’ follow-up non-invasively by comparing them with RECIST criteria.

## 2. Material and Methods

### 2.1. Sample Collection and Processing

We collected 12 mL of peripheral blood in EDTA vacutainer tubes from 20 metastatic CRC patients followed at the Department of Oncology at San Raffaele Hospital in Milan. All subjects gave their informed consent for inclusion before they participated in the study. The study was conducted in accordance with the Declaration of Helsinki and the protocol was approved by the Institutional Review Board of the San Raffaele Hospital (ctDNA/2017). Patients were enrolled consecutively by clinicians. The following clinical data were collected and reported in Table 1: primary colon cancer site, number and location of metastatic sites and therapy treatment.

The mutational status identified by the tissue biopsy was reported in Table 2. The biopsy on the tumour tissue was performed by the Department of Pathology at the San Raffaele Hospital by MassARRAY (Sequenom, San Diego, CA, USA), which is reported to provide a sensitivity of 5% in the detection of *RAS* and *BRAF* mutations.

Blood samples were taken at three different time points: T0 (before starting first-line treatment), T1 (corresponding to the first imaging evaluation of disease) and T2 (corresponding to the second imaging evaluation of disease). However, for patient number 2 it was not possible to continue collecting blood samples at T1 and T2 time points due to the patient’s death. Moreover, patient number 6 had decided to continue the therapy and follow-up in another hospital, closer to his home, while sample number 16 expressed a willingness to leave the study.

The evaluation of disease during treatment (T1 and T2) was performed by CT scan and according to RECIST (Response Evaluation Criteria in Solid Tumours) criteria version 1.1.

Plasma samples were separated as previously described [8] and were frozen at −20 °C. Circulating free DNA was extracted from 500 µL of plasma using the Maxwell RSC instrumentation (Promega, Madison, WI, USA) combined with the Maxwell^®^ RSC ccfDNA Plasma Kit (codice AS1480, Promega) and was eluted with 60 μL of Elution Buffer. For both the methodologies applied (microarray and ddPCR) we have performed a dedicated DNA extraction session, thus, for every sample, we have extracted two aliquots of plasma.

### 2.2. Microarray

#### 2.2.1. PCR Conditions

The DNA sequences containing the mutations were amplified using 5′-biotin forward and 5′-tagged reverse primers. Primer sequences, amplification length, annealing temperature (Ta) for the *KRAS* (codon 12-13, 61 and 146), *NRAS* (codon 12-13) and *BRAF* (codon 600) amplification, PCR reagents and cycling conditions were detailed in Reference [9]. 

In addition to the single amplification, we optimized a triplex PCR amplification in which the primers used for the *KRAS* codon 12-13, *NRAS* codon 12-13 and *BRAF* codon 600 were mixed together in the same PCR mixture. 

The triplex PCR was performed in 50 µL of reactions containing 15 µL of DNA extracted from plasma, 200 µM deoxynucleotide triphosphates, 10 mM Tris–HCl (pH 8.3), 50 mM KCl, 1.9 mM MgCl_2_, 1.95 U of DNA polymerase (FastStart Taq, Roche Diagnostics, Mannheim, Germany) and 20 pmoles of each primer with the exception of the *KRAS* codon 12-13 primer forward and reveres for which 40 pmoles were used.

Furthermore, to complete the panel of the remaining *KRAS* mutations, we also optimized a duplex PCR in which we co-amplify the *KRAS* codon 61 and codon 146 sequences at the same conditions reported above using 20 pmoles of primers for the *KRAS* codon 61 amplification and 40 pmoles for the *KRAS* codon 146 amplification.

#### 2.2.2. Microarray Preparation, Hybridization, Image Scanning and Data Analysis

Untreated silicon slides 1000A Thermal Oxide (14 mm × 14 mm) were supplied by Silicon Valley Microelectronics Inc. (Santa Clara, CA, USA). After an activation treatment (15 min) with oxygen plasma, the silicon chips were functionalized by dipping them for 30 min in solution (1% *w/v* in 0.9 M (NH_4_)_2_SO_4_ aqueous solution) of a copolymer of dimethylacrylamide (DMA), *N*-acryloyloxysucinimide (NAS) and meta-acryloy propyl trimethoxy silane (MAPS), copoly(DMA-NAS-MAPS)copoly with 10% *N*-acryloyloxysucinimide (NAS) moiety. This copolymer, commercially available under the trade name MCP-4 (Lucidant Polymers Inc. Sunnyvale, CA, USA) is an improvement of the copoly (DMA-NAS-MAPS) described in Pirri et al. [10] with an increased *N*-acryloyloxysucinimide (NAS) molar fraction (10% vs. 2%) to enhance its binding capacity. The copolymer forms a tri-dimensional functional coating on the surface of the silicon chips able to bind amino-modified oligonucleotide with high density. The chips were finally rinsed with water and dried under vacuum at 80 °C for 15 min.

The 5ʹ-end amino-modified oligonucleotides (capture probes), corresponding to thirteen *KRAS* mutations (G12A, G12C, G12D, G12R, G12S, G12V, G13D, Q61H(A>C), Q61H (A>T), Q61K, Q61L, Q61R and A146T), eight *NRAS* mutations (G12A, G12C, G12D, G12S, G12V, G13D, G13R and G13V), *BRAF* mutation (V600E), wild-type sequences (for sequences see Reference [2]) and a control oligonucleotide (COCU8), used as reference spots (Metabion International AG, Steinkirchen, Germany), were dissolved in the printing buffer (150 mM sodium phosphate pH 8.5, 0.01% Sucrose monolaurate) at a concentration of 10 µM and spotted on coated silicon chips by a piezoelectric spotter, SciFLEX ARRAYER S5 (Scienion, Berlin, Germany). Spotting was carried out at 20 °C in an atmosphere of 60% humidity. After the spotting step, the chips were incubated overnight and all residual reactive groups of the coating polymer were blocked as described in Damin et al. [11].

Prior to the hybridization of the spotted silicon chips, the biotinylated PCR products were thermally denatured to obtain single strands and purified by the use of streptavidin-coated magnetic beads (Dynabeads™ M-270 Streptavidin, Invitrogen). The ssPCRs were then incubated in a solution with a stabilizer, an oligonucleotide necessary to open the secondary structures present in the amplicon and were incubated for 10 minutes at room temperature. For the detection of wild-type and *KRAS, NRAS* and *BRAF* mutations, the reporters for the wild-type and the mutated sequences were added together in equimolar amounts to the tube containing the ssPCR-stabilizer solution. The incubation lasted for 35 minutes in gradient of temperature ranging from 42 °C to 29 °C.

A detailed description of the method was previously reported [12]. 

After the allele-specific hybridization occurred in solution, ssPCR-reporter hybrids were spread onto the spotted silicon chips and cover slips were placed on the spotted area. 

The chips were incubated at room temperature for 15 minutes in a humid hybridization chamber and then washed at room temperature in a 4× SSC buffer to remove the cover slip. This first wash step was followed by a brief wash (30 s) in a low-salt buffer (0.2× SSC). Then, to detect the mutations, the universal oligonucleotide labelled with Cy3 (Universal-Cy3) and a Cy3-labeled oligonucleotide (COCU10) complementary to COCU8, were mixed together in a solution at a 0.3 and 0.05 µM concentration respectively and spread onto the spotted silicon chips and cover slips were placed on the spotted area. The chips were incubated at room temperature for 15 min in a humid hybridization chamber. Finally, the silicon chips were removed from the hybridization chamber, washed and scanned as reported previously [12]. Fluorescence intensities were extracted with the scanner’s software (ScanArray Express, Version 4.0, PerkinElmer, Waltham, MA, USA, 2006) and the data analysis was performed for each experiment.

### 2.3. Droplet Digital PCR (ddPCR) 

The QX100™ Droplet Digital™ PCR System (Bio-Rad Laboratories, Hercules, CA, USA) instrument was used for this study. Eight µL of eluted DNA plasma sample were mixed with Commercial PrimePCR™ ddPCR™ Mutation Assays (assays ID were reported in Table S1 Supplemental data). ddPCR amplification reagents and cycling conditions were reported previously [8].

The fractional abundance of the mutated allele was calculated automatically by the QuantaSoft™ software version 1.7.4 (Bio-Rad) pooling results from the six fold and deriving fractional abundance of each mutation from a Poisson distribution. 

## 3. Results

### Microarray and ddPCR Analyses

**Figure 1:**
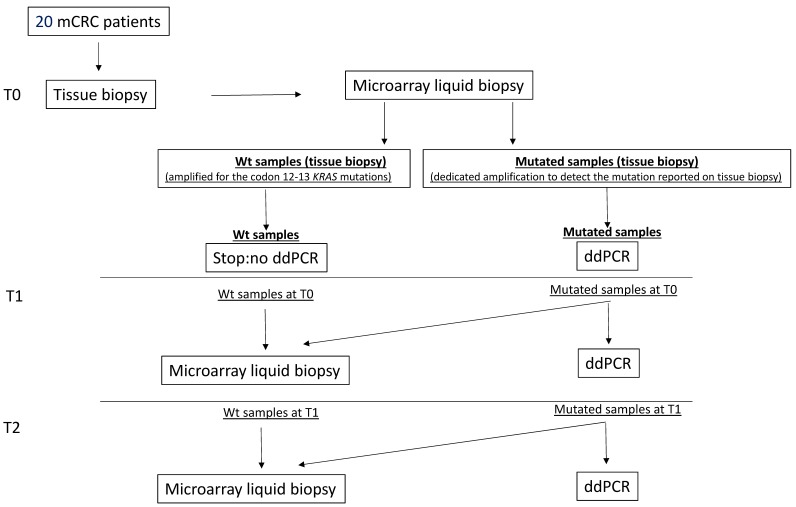
A complete scheme of the pipeline used in the study. T0, T1 and T2 = Time point 0, 1 and 2 respectively.

Considering the medical report of the tissue biopsy as a reference, we previously performed the mutational analysis in matched ctDNA plasma samples by microarray to identify the ctDNA mutation. For samples reported as wild-type on tissue biopsy only the more frequent mutations present on the *KRAS* codon 12-13 sequence were amplified while, for samples reported as mutated, a specific amplification to identify that definite mutation was performed. Then, to carry out a quantitative evaluation of the mutated allele, we performed a dedicated ddPCR using probes specific for the mutations characterized by the microarray platform. A complete scheme of the workflow used in this study at the time point T0, T1 and T2 was reported in Figure 1.

**Figure 2:**
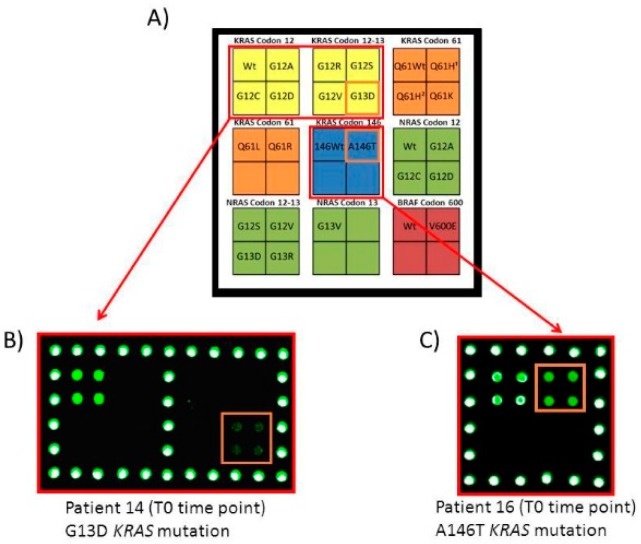
Microarray analysis of ctDNA extracted from plasma of patient 14 and 16 at T0 time point. (**A**) Schematic representation of the spotted barcode probe array. Each position in the grid identifies an individual barcode probe address corresponding to *KRAS* codon 12-13, *KRAS* codon 61 (Q61H1 c.183A>C, Q61H2 c.183A>T), *KRAS* codon 146, *NRAS* codon 12-13 and *BRAF* mutations. Silicon chips coated with copoly(DMA-NAS-MAPS) are used as substrates for the covalent attachment of amino-modified barcode probe oligonucleotides arrayed at discrete locations. The white portion of the array is spotted with an amino-modified oligonucleotide (COCU8), not correlated with the genes, to be used as reference spots. (**B**) Microarray scanning of the Cy3 fluorescence signal of the analysis of ctDNA extracted from plasma of patient 14 at T0 time point. Only the part of the array corresponding to the barcode probes for *KRAS* codon 12 and 13 is shown. The robotically spotted array is hybridized with an individual single strand PCR incubated with the whole set of dual-domain reporters. The fluorescence detection is obtained incubating the array with a mixture of the universal Cy3 labelled oligonucleotide complementary to the tagged-reverse primer of the single strand PCR and with a Cy3-labeled oligonucleotide (COCU10) complementary to COCU8. The barcode probes corresponding to *KRAS* G13D is highlighted. (**C**) Microarray scanning of the Cy3 fluorescence signal of the analysis of ctDNA extracted from plasma of patient 16 at T0 time point. Only the part of the array corresponding to the barcode probes for *KRAS* codon 146 is shown. The frame highlights the barcode probes corresponding to the *KRAS* A146T mutation.

The results obtained analysing by microarray all the 20 samples at the T0 time point are reported in Table 2, while, in Figure 2, microarray images of the analysis performed on two patients plasma samples with low (n.14) and high (n.16) fluorescence signals corresponded to *KRAS* G13D and A146T mutations respectively, were shown. 

The overall concordance between microarray and tissue biopsy was 95% (19 samples out of 20). Indeed, the microarray analyses identified the G12C *KRAS* mutation in sample n.7, consequently confirmed by the ddPCR approach that had not been reported in tissue biopsy medical report.

**Figure 3:**
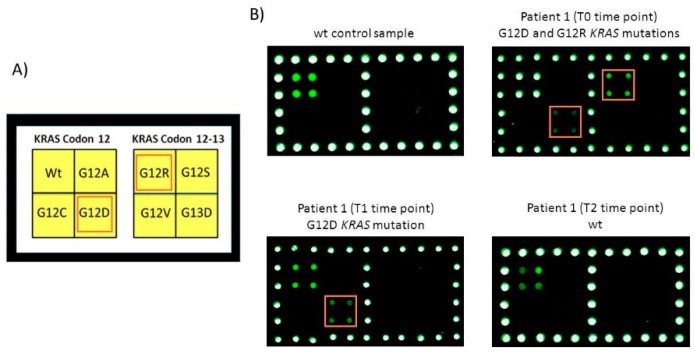
Analysis of ctDNA extracted from plasma of patient 1 at T0, T1 and T2 time points. (**A**) Spotting schema of the portion of the barcode probe array reported in Figure 2A corresponding to the barcode probes for *KRAS* codon 12 and 13. (**B**) Cy3 fluorescence images of the analysis of ctDNA extracted from a control wild-type plasma sample and from plasma of patient 1 at T0, T1 and T2 time points. The barcode probes corresponding to *KRAS* G12D and G12R are highlighted.

Moreover, besides that described in tissue biopsy, the microarray revealed a second mutation (*KRAS* G12R) in sample n.1 (Figure 3, T0 time point). 

Nine out of 20 samples were subsequently analysed by ddPCR using the probes specific for the mutations identified by the microarray analysis (Table 2). The fractional abundance of the mutated allele ranged from 0.033 to 77.5%.

For the remaining 11 samples (55%) reported as wild-type by microarray no further analysis were performed by ddPCR.

For 12 out of 20 patients we obtained the plasma sample also at T1 time point and for 5 out of 20 even at T2 time point. The results obtained were reported in Table 3 together with the clinical evaluation based on RECIST criteria. For 10 out of 12 samples were reported a “stable disease” or partial/complete response consistent with the results obtained by liquid biopsy. 

**Figure 4:**
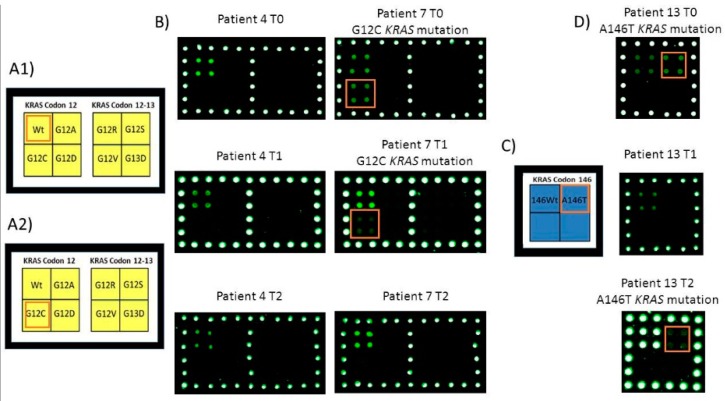
Analysis of ctDNA extracted from plasma of patient 4, 7 and 13 at T0, T1 and T2 time points. (**A1**,**A2**) Spotting schema of the portion of the barcode probe array reported in Figure 2A corresponding to the barcode probes for *KRAS* codon 12 and 13. (**B**) Cy3 fluorescence images of the analysis of ctDNA extracted from plasma of patient 4 and 7 at T0, T1 and T2 time points. The barcode probes corresponding to *KRAS* G12C in patient 7 are highlighted; the patient 4 is *KRAS* wild-type. (**C**) Spotting schema of the portion of the barcode probe array reported in Figure 2A corresponding to the barcode probes for *KRAS* codon 146. (**D**) Cy3 fluorescence images of the analysis of ctDNA extracted from plasma of patient 13 at T0, T1 and T2 time points. The barcode probes corresponding to KRAS A146T in patient 13 are highlighted.

In Figure 3 and Figure 4 some examples of the microarray results obtained from the same patient in whole time point T0, T1, T2 were shown. In particular, for sample number 1 (Figure 3) at T1 time point only the *KRAS* G12D mutation was still present while, at T2 time point, no mutations were identified. In Figure 4, microarray’s images of the analysis of samples number 4 (wild-type sample), 7 and 13 (mutated *KRAS* G12C and A146T samples, respectively) were shown. Sample number 7 showed, at T1 time point, a reduction of the fluorescence corresponded to the *KRAS* G12C mutation in agreement with the clinical evaluation that reported a partial response while, at T2 time point, no mutation signal was present in accordance to the RECIST classification of complete response. 

Concerning sample number 13 after a partial response at T1 time point that corresponded a no signal of the driver mutation by microarray analysis, at T2 time point, the mutation signal returned to be again detectable in agreement with the clinical evaluation that reported a progression of the disease.

**Figure 5:**
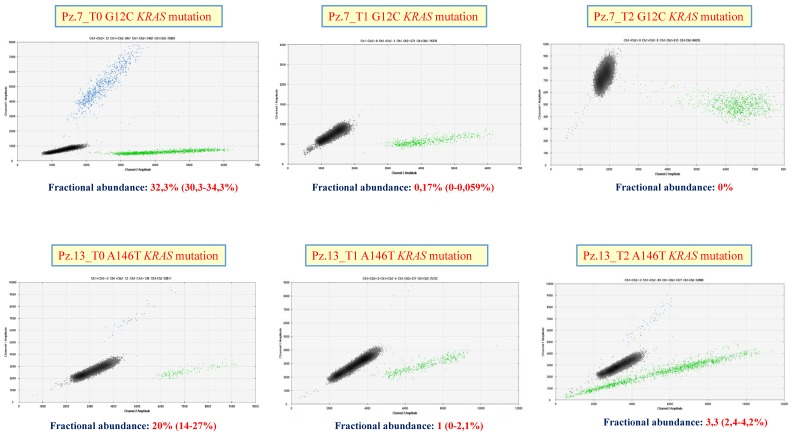
Droplet digital PCR analysis. Examples of ddPCR plot for two metastatic colorectal cancer (mCRC) patients (n.7 and n.13) and respective fractional abundance of the mutated *KRAS* allele at the 3 time point: Patient n.7 = G12C *KRAS* mutation (upper panel) and Patient n.13 = A146T *KRAS* mutation (lower panel). Mutants are clustered in the upper left corner with high Fluorescein (FAM) fluorescent intensities (blue), wild-type is clustered in lower right corner with high Hexachloro-fluorescein (HEX) fluorescent intensities (green) while mutant plus wild-type sequences are clustered in the upper right corner (orange). The empty droplets are represented in black.

In Figure 5, the corresponding ddPCR analyses of samples n.7 and n.13 were shown. For sample number 7 a consistent decreased of the fractional abundance of the mutated allele was revealed at T1 time point until to be absence at the T2 time point, while for sample number 13, after an initial decreased of the fractional abundance at T1 time point (from 20% up to 1%) an increment of the mutated allele was detectable at subsequently T2 time point (3.3%).

We observed a complete concordance with the clinic report that considered these patients with “Complete Response” and “Progression Disease,” respectively.

**Figure 6:**
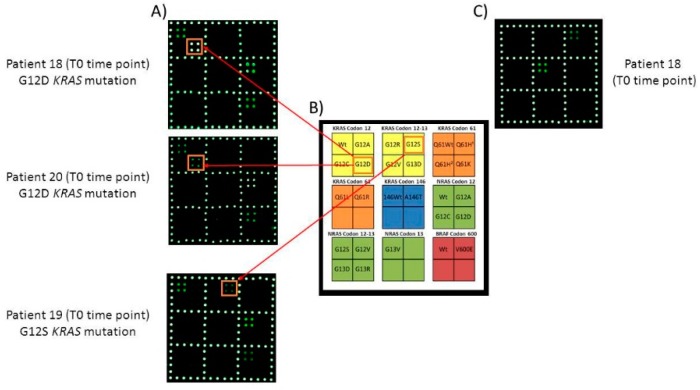
Multiplex analysis of ctDNA extracted from plasma of patient 18, 19 and 20 at T0 time point. (**A**) Cy3 fluorescence images of the triplex analysis (with all the reporters for *KRAS* and *NRAS* codon 12-13 and *BRAF*) of ctDNA extracted from plasma of patients 18, 19 and 20 at T0 time point. The barcode probes corresponding to *KRAS* G12D (patients 18 and 20) and G12S (patient 19) are highlighted. (**B**) Schematic representation of the spotted barcode probe array. Q61H^1^ c.183A>C, Q61H^2^ c.183A>T. (**C**) Cy3 fluorescence image of the duplex analysis (with all the reporters for *KRAS* codon 61 and 146) of ctDNA extracted from plasma of patient 18 at T0 time-point. Only the barcode probes corresponding to *KRAS* codon 61 and 146 wild-type sequences are shown.

Moreover, to assess the possibility of using the microarray even without previously knowledge the medical report of tissue biopsies, we performed two multiplex PCR. Indeed, we optimized a triplex PCR that includes the most frequent mutations in mCRC patients (*KRAS* and *NRAS* codon 12-13 and *BRAF* V600E mutations) and a duplex in which the remaining *KRAS* mutations (codon 61 and 146) were included, to be able, with only two runs of amplification, to cover all the 22 mutations identifiable by the microarray. The two multiplex PCR were performed for samples n.18, 19 and 20 and results were shown in Figure 6. Also applying the triplex amplification, the *KRAS* G12D and G12S mutations were detectable in patients n.18, 20 and 19 respectively, with no loose of sensitivity and specificity of the microarray approach.

## 4. Discussion

In cancer management, the mCRC patients were subjected to tissue biopsy to specifically highlight *RAS* and *BRAF* mutational status (in patients with *RAS* wild-type) as key biomarkers to be assayed at the time of diagnosis of stage IV disease as recommended by the Current National Comprehensive Cancer Network guidelines. It is now established that assessing ctDNA in the plasma of cancer patients is a promising process to evaluate somatic mutations from solid tumours (especially in metastatic phase) in a non-invasive manner.

Since the rapid and incremental improvements in instrumentations, several methodologies have been investigated so far among which NGS and ddPCR seem to be the more focused for liquid biopsy [13,14]. However, the NGS approach is too expensive (even if we consider a targeted approach) and required dedicated technical expertise that appears more appropriated in a research field rather than integrated into a clinical setting. Moreover, in the literature it is reported that, in mCRC patients with oligometastatic disease, NGS on cfDNA was feasible but had limited sensitivity to detect somatic mutations while ddPCR and mutant allele enrichment before NGS appeared to be more sensitive [15]. On the contrary, the ddPCR approach is limited to analysing a single mutation for each run making its use unsuitable even if we consider the possibility of performing multiplex assays while remaining the quantitative technique of choice for the assessment of the fractional abundance of the mutation.

We described a microarray platform for specific, sensitive and rapid detection of the most common mutations in the *RAS* (*KRAS* and *NRAS*) and *BRAF* genes suitable for high-throughput analysis without expensive instrumentation by maintaining accuracy and robustness.

In our approach, the ddPCR is used downstream the microarray that previously identifies in a non-expensive, simple to handle and fast way the mutation involved. 

Also, the amount of plasma required in our pipeline (1mL) is compatible with clinical management.

By this combined approach, our results showed 95% of overall concordance with those reported by tissue biopsy. In the plasma of sample number 7 we detected a mutation (*KRAS* G12C) absent in the tissue biopsy report. This could be ascribed to the limitation we have in genotyping CRC tissue; a tissue sample represents a single photograph in time and it is subjected to spatial selection bias owing to intratumor heterogeneity and metastatic stadiation. However, the treatment received by the patient (Folfox and Panitumumab) was able to produce a complete response of the disease. We can hypothesize that the *KRAS* G12C mutation could be present in a non-preponderant tumour subclone. Nevertheless, it could be extremely important to non-invasively monitor the presence of this mutation in the future to decide eventually a change of the treatment provided. 

Ghatalia et al. reported the same phenomenon [16]. In a patient where two serial liquid biopsy were performed both revealing the *KRAS* G12D mutation, the tissue biopsy obtained at a time point between the two blood drawn did not reveal *RAS* mutations. 

Also, the double mutation detected by the microarray in patient number 1 could be due to intrinsic factors such as tumour clonal heterogeneity. In this case, no change of treatment was necessary; however, to identify a second mutation in plasma is fundamental to monitor the therapeutic response over time. Indeed, at the time point T1 and T2 we were able to monitor the trends of both mutations and only the mutation not detected by tissue biopsy is the one that persists over time even if in a reduced amount. This was in agreement with was reported by CT scan that identified a reduction of the disease at time point T1 and stable disease at time point T2. 

For 12 out of 20 samples, we also tested the plasma at the T1 time point and for 5 out of the 12 samples also at the T2 time point obtaining an overall concordance between liquid biopsy and clinical follow-up. As an example the clinical history of the patient number 7 at the moment of the T2 blood drawing was reported as “complete response” and no ctDNA was found in plasma while for the patient number 13 after a “disease in regression” reported in T1 (which corresponds to the absence of ctDNA) reported an “increase of the disease” at the T2 time point corresponding at an increase up to 3.3% of the mutated allele.

The ctDNA concentration correlates with disease staging and progression with the exception of sample number 19 in which at the T1 time point the ddPCR registered a fractional abundance of the mutated allele similar to that reported at the T0 time point (stable disease) while by CT scan a progression of the disease was showed. However, no therapy changes were decided for the patient but a closer outpatient visit. In this study, we also reported the death of patient number 2 that presented the V600E *BRAF* mutation and a right-sided colon cancer both indicators of worse prognosis [17,18].

All the wild-type patients for *RAS* and *BRAF* mutations have remained wild-type also at the T1 and T2 time point. Nevertheless, it is crucial to monitor them over time to reveal developing subclonal resistant mutations acquisition as a known mechanism of secondary resistance in mCRC patients receiving anti-EGFR therapy [19]. 

In conclusion, the combined approach here presented is fast (using the tag-microarray method, the genotyping of clinical sample can be obtained in less than 90 min) a time significantly shorter even if we consider it combined subsequently with the ddPCR approach. The current gold standard procedure for clinical diagnosis normally takes 1–2 working days. 

Moreover, even the multiplex amplification allowed us to correctly identify the mutations in samples 18, 19 and 20, previously tested by single amplification, without loss in sensitivity and specificity.

However, it is correct underline that in these three samples the fractional abundance of the mutated allele reported were conspicuous. Thus, we need to validate the multiplex PCR approach also in samples with a very low ctDNA amount to be sure to avoid missing the identification of the mutation. Nevertheless, it is a promising method that could allow us to analyse 22 mutations performing only two PCR session. 

By applying a dedicated analytical methodology ctDNA could be a reliable non-invasive biomarker for *RAS* and *BRAF* mutations in mCRC patients allowing for the identification of the target mutation and to monitor the disease load to provide clinically relevant information as supported by researchers and experts attended at the American Society of Clinical Oncology (ASCO) 2018 [20].

In the forthcoming future, it could be also interesting develop assays to identify the *PI3KCA* mutations finding new insights regarding clinical settings [21,22].

We believe that integrating ctDNA in mCRC patient management could provide a whole and real-time assessment of the cancer mutation status.

## Figures and Tables

**Table 1:** Clinical data of the patients enrolled in the study.

Sample ID	Tissue Biopsy	Sidedness of the Primary Tumour	Number and Location of Metastatic Sites	First-Line Treatment
1	*KRAS* G12D	descending colon	2 (liver, lung)	FOLFOX + BEVA
2	*BRAF* V600E	ascending colon	3 (stomach, peritoneum, lymph nodes)	Not applicable: performance status deterioration
3	wt	sigmoid colon	1 (lung)	FOLFOX + PANI
4	wt	sigmoid colon	2 (liver, lymph nodes)	FOLFOXIRI + PANI
5	wt	sigmoid colon	1 (liver)	FOLFOX + PANI
6	wt	rectum	2 (lung, liver)	FOLFOX + BEVA
7	wt	splenic flexure, sigmoid colon-rectum	1 (liver)	FOLFOX + PANITUMUMAB
8	wt	descending colon	1 (peritoneum)	FOLFIRI + CETUXIMAB
9	wt	ascending colon	2 (lung, liver)	CAPECITABINA
10	wt	rectum	1 (lung)	FOLFIRI + CETUXIMAB
11	wt	sigmoid colon	2 (liver, peritoneum)	FOLFOX + CETUXIMAB
12	wt	transverse colon	1 (liver)	FOLFOX + PANI
13	*KRAS* A146T	descending colon	3 (liver, peritoneum, lymph nodes)	XELOX + BEVA
14	*KRAS* G13D	sigmoid colon	2 (liver, lymph nodes)	FOLFOX + BEVA
15	wt	descending colon	1 (liver)	FOLFOX + CETUXIMAB
16	*KRAS* A146T	cecum	2 (right iliac bone, muscles)	XELOX+BEVA
17	wt	cecum, rectum	1 (peritoneum)	FOLFOX + PANITUMUMAB
18	*KRAS* G12D	hepatic flexure	1 (liver)	FOLFOX+BEVACIZUMAB
19	*KRAS* G12S	descending colon	1 (liver)	FOLFOX+BEVACIZUMAB
20	*KRAS* G12D	rectum	1 (liver)	FOLFOX+BEVACIZUMAB

**Table 2:** Patients enrolled and corresponding mutational analysis on tissue biopsy and liquid biopsy at the T0 time-point by microarray and ddPCR approaches.

Sample ID	Tissue Biopsy	Liquid Biopsy	
		Microarray	ddPCR Mutated Allele %
1	*KRAS* G12D	*KRAS* G12D + G12R	*KRAS* G12D = 0.033 *KRAS* G12R = 0.95%
2	*BRAF* V600E	*BRAF* V600E	*BRAF* V600E = 15%
3	wt	wt	-
4	wt	wt	-
5	wt	wt	-
6	wt	wt	-
7	wt	*KRAS* G12C	*KRAS* G12C = 32.3%
8	wt	wt	-
9	wt	wt	-
10	wt	wt	-
11	wt	wt	-
12	wt	wt	-
13	*KRAS* A146T	*KRAS* A146T	*KRAS* A146T = 20%
14	*KRAS* G13D	*KRAS* G13D	*KRAS* G13D = 1.3%
15	wt	wt	-
16	*KRAS* A146T	*KRAS* A146T	*KRAS* A146T = 77.5%
17	wt	wt	-
18	*KRAS* G12D	*KRAS* G12D	*KRAS* G12D = 65.3%
19	*KRAS* G12S	*KRAS* G12S	*KRAS* G12S = 17.7%
20	*KRAS* G12D	*KRAS* G12D	*KRAS* G12D = 3.7%

**Table 3:** Comparison of liquid biopsy results from patients’ enrolment at T1 and T2 and clinical evaluation.

Sample ID	Microarray	ddPCR Mutated Allele %	Clinical Evaluation RECIST Classification
Pz 1 T0 Pz 1 T1 Pz 1 T2	G12D + G12R G12D wt	G12D = 0.033%; G12R = 0.95% G12D = 0.4 %; G12R = 0.22% G12D = 0%; G12R = 0.25%	>> PRSD
Pz 3 T0 Pz 3 T1 Pz 3 T2	wt wt wt	- - -	>>PR CR
Pz 4 T0 Pz 4 T1 Pz 4 T2	wt wt wt	- - -	>>PR CR
Pz 5 T0 Pz 5 T1	wt wt	- -	SD
Pz 7 T0 Pz 7 T1 Pz 7 T2	G12C G12C wt	G12C = 32.3% G12C = 0.17% G12C = 0%	>>PR CR
Pz 10 T0 Pz 10 T1	wt wt	- -	SD
Pz 11 T0 Pz 11 T1	wt wt	- -	SD
Pz 13 T0 Pz 13 T1 Pz 13 T2	A146T wt A146T	A146T = 20% A146T = 1% A146T = 3.3%	>>PR PD
Pz 14 T0 Pz 14 T2	G13D G13D	G13D = 1.3% G13D = 0.22%	>>PR
Pz 17 T0 Pz 17 T1	wt wt	- -	SD
Pz 19 T0 Pz 19 T1	G12S G12S	G12S = 17.7% G12S = 14%	PD
Pz 20 T0 Pz 20 T1	G12D G12D	G12D = 3.7% G12D = 0.3%	PR

PR = Partial Response, SD = Stable Disease, CR = Complete Response; PD = Progression Disease.

## Supplementary Materials

The following are available online at https://www.mdpi.com/2073-4409/8/8/769/s1, Table S1: Supplemental data. Specific ddPCR Assay ID.
