# Supplementary material for: Microarray Approach Combined with ddPCR: An Useful Pipeline for the Detection and Quantification of Circulating Tumour DNA Mutations
Source: Cells. 2019 Jul 24;8(8):769. doi: 10.3390/cells8080769 (PMC6721623; doi:10.3390/cells8080769)
Supplement: Supplementary file 1 [file cells-08-00769-s001.pdf]

Table 1 Supplemental data. Specific ddPCR Assay ID.

Gene	Nucleotide Mutation	Aminoacid change	Assay ID (wild-type sequence)	Assay ID (mutated sequence)
KRAS (exon 2)	c.34G>T	p.G12C	dHsaCP2500585	dHsaCP2500584
	c.35G>A	p.G12D	dHsaCP2500597	dHsaCP2500596
	c.34G>C	p.G12R	dHsaCP2500591	dHsaCP2500590
	c.34G>A	p.G12S	dHsaCP2500589	dHsaCP2500588
	c.38G>A	p.G13D	dHsaCP2500599	dHsaCP2500598
KRAS (exon 4)	c.436G>A	p.A146T	dHsaCP2000080	dHsaCP2000079
BRAF (exon 15)	c.1799T>A	p.V600E	dHsaCP2000028	dHsaCP2000027
